# What is the best dialysis regimen for severe AKI to allow kidney function recovery?

**DOI:** 10.1093/ckj/sfag278

**Published:** 2026-08-17

**Authors:** Yu-Ting Chou, Charles Jun Han Ng, Vin-Cent Wu, Dmitry Sokolov, Turgay Saritas, Marlies Ostermann

**Affiliations:** Division of Nephrology, Department of Internal Medicine, National Taiwan University Hospital, Taipei, Taiwan; Department of Renal Medicine, Changi General Hospital, Singapore; Department of Intensive Care,King’s College London, Guy’s & St Thomas’ NHS Foundation Trust, London, UK; Division of Nephrology, Department of Internal Medicine, National Taiwan University Hospital, Taipei, Taiwan; Department of Anesthesiology and Intensive Care, First Pavlov St. Petersburg State Medical University, Petersburg,Russia; Department of Nephrology, University Hospital RWTH Aachen, Aachen, Germany; Department of Internal Medicine, Nephrology and Transplantation, Erasmus Medical Center Rotterdam, Rotterdam,The Netherlands; Department of Intensive Care,King’s College London, Guy’s & St Thomas’ NHS Foundation Trust, London, UK

**Keywords:** acute kidney injury, dialysis, dialytrauma, renal recovery

## Abstract

Acute kidney injury (AKI) recovery is a major determinant of long-term renal and patient outcomes. While transient AKI is associated with a more favorable prognosis, persistent or incomplete recovery increases the risk of chronic kidney disease, dialysis dependence, cardiovascular complications, and mortality. Renal recovery after AKI reflects the balance between adaptive repair processes and maladaptive pathways leading to fibrosis and nephron loss. Experimental and clinical evidence highlights the roles of tubular epithelial regeneration, macrophage phenotypes, inflammatory mediators, complement activation, and aging-related decline in regenerative capacity in determining recovery trajectories. Recurrent injury and persistent hemodynamic stress may further impair renal repair and promote progression to chronic kidney disease. Renal replacement therapy (RRT) may itself contribute to delayed renal recovery through “dialytrauma”, a concept that encompasses all harmful adverse events related to RRT. Major mechanisms of dialytrauma include hemodynamic instability and organ ischemia, blood–membrane inflammatory activation, metabolic and osmotic perturbations, and access-related complications. Intermittent hemodialysis is particularly associated with circulatory and osmotic stress, whereas continuous RRT may expose patients to prolonged extracorporeal circulation, anticoagulation-related complications, and cumulative metabolic derangements. Peritoneal dialysis avoids extracorporeal blood contact but has other potential risks. This review summarizes the current understanding of dialytrauma as a potentially modifiable determinant of nonrecovery of renal function in patients with AKI receiving RRT. No single modality is universally superior, and minimizing unnecessary exposure, excessive intensity, and hemodynamic instability is essential. In this article, we discuss strategies to minimize the risk of dialytrauma to guide individualized RRT strategies with the aim to promote renal recovery and preserve long-term kidney health.

## INTRODUCTION

Acute kidney injury (AKI) is a multifactorial syndrome with a broad pathophysiology and diverse outcomes [1–3]. At the severe end of the AKI spectrum, dialysis-requiring AKI (AKI-D) is associated with particularly poor renal outcomes. Recovery of renal function is defined as resolution of AKI, which may be partial or complete. Recovery within 48 h (i.e. transient AKI) has been associated with better renal and nonrenal outcomes compared to persistent AKI lasting for >48 h [4]. Further, patients with partial or no recovery of kidney function after AKI have a higher risk of developing chronic kidney disease (CKD), long-term dialysis dependence, cardiovascular morbidity, cerebrovascular disease, and rehospitalization [5]. Several factors contribute to nonrecovery, some of which are irreversible (e.g. pre-existing CKD) and others are potentially modifiable (e.g. intra-dialytic hypotension), but many aspects are still unknown. In general, prevention of AKI-D and correction of the underlying cause of AKI are among the most important strategies to promote renal recovery.

This review summarizes some of the known mechanisms of resolution of renal function after AKI-D and outlines the concept of “dialytrauma”, a collective term for all adverse events related to renal replacement therapy (RRT).

## BIOLOGICAL MECHANISMS DURING RENAL RECOVERY

In AKI, distinct patterns of tubular cell death have been observed, including cellular apoptosis, ferroptosis, or necrosis. The release of chemotactic factors like chemokine C-C motif chemokine ligand 14 (CCL14) by damaged epithelial cells promotes intrusion of monocytes into the renal interstitial compartment and transformation to macrophages [6]. Distinct macrophage subsets exist, with some having predominantly pro-inflammatory properties and others contributing to tubular epithelial regeneration and attenuation of inflammation [7].

The transition from AKI to complete recovery versus progression to CKD appears to be determined by the balance between adaptative and maladaptive repair processes. Adaptive tubular repair is thought to depend primarily on the capacity of surviving injured tubular epithelial cells to undergo dedifferentiation, proliferation, and redifferentiation, rather than on a fixed pool of pre-existing immature cells [8]. In the glomerulus, CD133+/CD24+ parietal epithelial cells have shown progenitor potential, and experimental lineage-tracing studies suggest that parietal epithelial cells can contribute to podocyte replacement in selected disease contexts.

Cell cycle arrest and proinflammatory mediators (i.e. MCP1 and CCL2) can be protective in the early phase of kidney injury. However, if prolonged, fibrosis may set in, resulting in loss of kidney function. Transforming growth factor beta 1 (TGF-b1) is produced by renal resident cells as well as infiltrating immune cells (macrophages, lymphocytes) and plays an important role in activating fibroblasts and increasing collagen production. Complement activation also contributes to nonrepair of renal tissue [9]. Further, repeated episodes of renal injury prevent tissue regeneration and decrease the likelihood of sustained recovery [10, 11].

Aging can significantly alter the regenerative capacity of the kidney. Diminished proliferative reserve, decreased functional adaptability of macrophages, and an increase in the expression of TGF- b1 represent distinct processes that explain why older kidneys are more prone to maladaptive repair after AKI [12]. The nephrons that remain following kidney injury often undergo structural hypertrophy, resulting in increased glomerular filtration (“single nephron hyperfiltration”) [13]. Persistent hyperfiltration can eventually lead to a vicious circle of proteinuria, glomerulosclerosis, further reduction of renal functional reserve, and increased susceptibility to recurrent AKI.

## DIALYTRAUMA

RRT can be lifesaving but also has side effects depending on the modality and application (Table 1 and Fig. 1). The term “dialytrauma” encompasses all adverse events related to RRT, some of which may impact renal recovery [14, 15]. A pragmatic way to understand the mechanisms of dialytrauma (Fig. 1) is to differentiate between (i) hemodynamic instability and organ ischemia, (ii) biocompatibility and inflammatory activation, (iii) metabolic perturbations, and (iv) access-related complications [14, 16–19].

**Figure 1:**
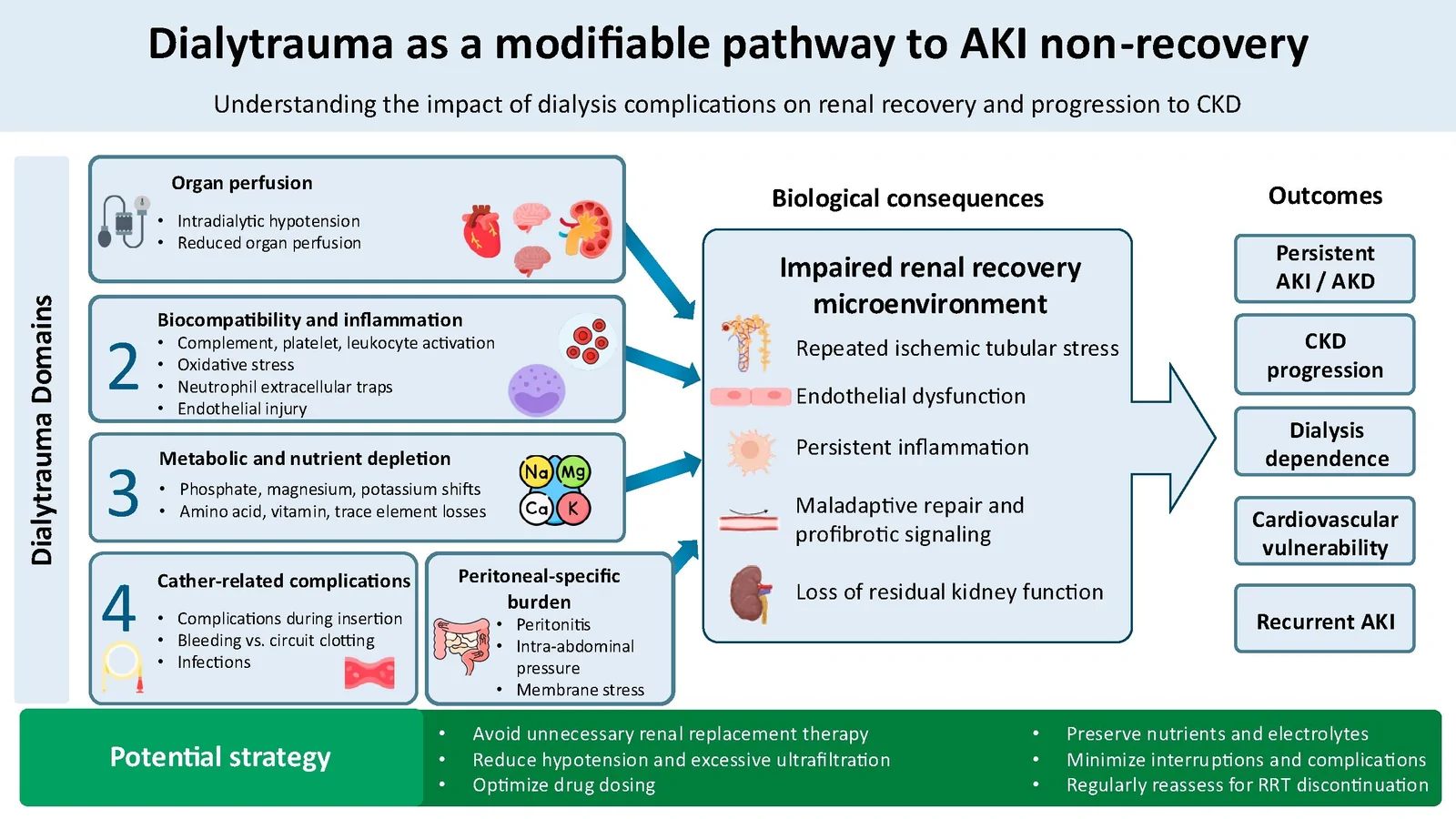
Dialytrauma as a modifiable pathway to AKI nonrecovery. The figure summarizes the key pathophysiological processes and biological consequences that are associated with reduced chances of resolution of acute kidney injury and nonrecovery of renal function.

**Table 1:** Characteristics of different renal replacement therapy modalities used for AKI.

Factor	Modality			
	Intermittent hemodialysis	Hybrid RRT SLED/EDD/PIRRT	CRRT	PD
Duration (h)	4–6	6–16	24	24
Frequency	daily/alternate days	daily/alternate days	daily	daily
Mechanism of solute transport	Diffusion ± convection	diffusion, convection or both	diffusion, convection or both	diffusion
Blood flow (ml/min)	200–350	100–300	100–250	not applicable
Main access	central venous dialysis catheter	central venous dialysis catheter	central venous dialysis catheter	peritoneal dialysis catheter
Need for anticoagulation	usually but not absolutely necessary	usually but not absolutely necessary	usually but not absolutely necessary	no
Fluctuations of osmotically active metabolites	++	+	less but may occur in case of treatment interruptions	no
Fluctuations of intravascular volume status	++	+	less but may occur in case of treatment interruptions	no
Effect on plasma concentrations of renally cleared drugs	major fluctuations	some fluctuations	less fluctuations	less fluctuations

ICP, intracerebral pressure; RRT, renal replacement therapy; SLED, sustained low-efficiency dialysis; EDD, extended daily dialysis; PIRRT, prolonged intermittent renal replacement therapy.

### Hemodynamic instability and organ ischemia

Hemodynamic instability is often due to a mismatch between fluid removal and the patient’s ability to maintain an effective circulating volume and organ perfusion. When fluid removal exceeds plasma refilling, venous return decreases, myocardial dysfunction may develop, and the ability to maintain adequate tissue perfusion declines [20, 21]. Clinically, this often manifests as hemodynamic instability, potentially reduced myocardial and cerebral perfusion, and interruptions in RRT [20–22]. Repeated episodes of intradialytic hypotension may expose the recovering kidney to recurrent ischemic stress, particularly in the renal cortex and outer medulla, which could plausibly interfere with repair. However, direct evidence that intradialytic hypotension independently leads to renal nonrecovery in AKI-D remains limited, as most clinical data are observational and confounded by illness severity and indication for RRT. Beyond excessive ultrafiltration, high-intensity RRT may also induce significant hemodynamic changes not only by affecting preload but also through fluctuations of systemic vascular resistance and temperature effects on vascular tone [23].

Human imaging studies have provided direct evidence of intradialytic hypoperfusion during intermittent RRT. Cerebral positron emission tomography (PET) with near-infrared spectroscopy revealed reductions in cerebral blood flow and oxygenation during intermittent hemodialysis (HD) that were not fully explained by changes in systemic blood pressure, implicating impaired autoregulation and increased microvascular vulnerability [24, 25]. Similar findings were also seen in both kidneys and the heart. CT perfusion studies have quantified acute reduction in renal perfusion during intermittent HD, which correlated with markers of myocardial stunning [25]. Similarly, cardiac perfusion imaging and echocardiographic functional measures have shown links between intradialytic circulatory stress and myocardial perfusion deficits and stunning [26]. Multiparametric intradialytic magnetic resonance imaging (MRI) has extended this framework beyond single-organ endpoints by capturing concurrent changes in cerebral arterial flow and renal structural signals during chronic HD. These findings support the notion that dialysis-associated circulatory stress is a multi-organ phenomenon rather than an isolated blood pressure problem [27].

### Biocompatibility and inflammatory activation

Blood–membrane and circuit interactions contribute to dialytrauma [28, 29]. When blood is exposed to an extracorporeal circuit, surface-driven immunecoagulation may promote microinflammation, thrombogenicity, platelet consumption, and vasoactive reactions [28–30]. This cascade can start within seconds of plasma proteins being adsorbed by the membrane and circuit, triggering platelet adhesion and activation with thrombin generation [31, 32]. Complement activation releases C3a/C5a and terminal pathway signals that amplify leukocyte activation and oxidative/inflammatory injury [29, 33]. Further, neutrophil activation followed by degranulation and formation of neutrophil extracellular traps (NETs) contribute to a proinflammatory and prothrombotic environment [34]. In parallel, artificial surfaces can trigger the contact activation pathway system, which activates FXII to FXIIa, linking intrinsic coagulation, kallikrein–kinin signaling (bradykinin), and inflammation [33]. These intermittent inflammatory hits provide a biologically plausible pathway that may influence the renal repair microenvironment. Nevertheless, whether this translates into impaired kidney recovery and development of fibrosis following AKI-D requires further research.

### Metabolic perturbations

Metabolic dialytrauma arises when solute, electrolyte, and buffer shifts outpace physiological adaptation. Acute acid–base disturbances may alter the renal recovery microenvironment and impair the clearance of protein-bound uremic toxins [35]. Abrupt reductions in plasma urea and osmolality can also precipitate dialysis disequilibrium syndrome through brain–plasma osmotic gradients and cerebral edema, particularly at dialysis initiation in patients with advanced CKD; preventive approaches favor gentler initial clearance and sodium or solute profiling, although the supporting evidence remains limited [36, 37]. At the same time, RRT can deplete phosphate, potassium, and magnesium, while also removing essential amino acids, water-soluble vitamins, and trace elements, thereby contributing to protein–energy wasting and micronutrient deficiency [38–43]. While these nutritional losses are potentially clinically relevant, there is limited evidence that monitoring or replacement of specific nutrients or micronutrients improves renal recovery. In selected settings, hemolysis byproducts and free heme or iron may impose an additional nephrotoxic burden through tubular toxicity, luminal obstruction, and intrarenal inflammation [44].

### Access-related complications

Access-related injury contributes to dialytrauma through procedure-related complications and cumulative risks, including mechanical injury, infection, and treatment interruptions during periods of access dysfunction [45–47]. Adverse effects of anticoagulation, coupled with catheter-related complications (infection, thrombosis, and venous stenosis), may lead to systemic inflammation, bleeding complications, and hemodynamic instability, potentially resulting in prolonged critical illness and delayed renal recovery.

## TYPES OF RRT AND RISK OF DIALYTRAUMA

RRT modalities vary in their characteristics (Table 1) and can contribute to dialytrauma through distinct dominant injury patterns and should be regarded as distinct exposures (Table 2). Understanding of the distinct physiological and clinical stressors associated with each modality may inform individualized management tailored to the patient’s specific condition. Intermittent HD tends to generate more physiological fluctuations in fluid status, serum osmolality, and occasionally hemodynamics compared to continuous RRT (CRRT) [20, 21, 30]. On the other hand, CRRT is associated with more prolonged exposure to extracorporeal circuits, anticoagulation trade-offs, and potentially some cumulative metabolic drifts. Peritoneal dialysis (PD) avoids extracorporeal blood contact and hemoincompatibility but is associated with acute peritoneal membrane stress [28, 48–50]. Studies comparing different modalities have had heterogeneous results depending on patient characteristics and study protocols (Table 3).

**Table 2:** Types of RRT and risk of dialytrauma.

RRT modality	Dialytrauma focus	Pathophysiological mechanisms	Hemodynamic impact	Risk profile	Clinical implication
Intermittent HD	Circulatory stress and repetitive ischemic end-organ damage	Rapid fluid and solute removal leading to volume-perfusion mismatch and transient osmotic shifts. Contact with the extracorporeal circuit activates clotting cascade, complement, and inflammation.	Known: High incidence of intradialytic hypotension and recurrent organ stunning Probable: acute decline in MAP and reduction in perfusion to the heart, brain, and kidneys.	Hemodynamics: High Biocompatibility: High Metabolic: Moderate	May be more tolerable in hemodynamically stable patients.
CRRT	Extensive nutritional and micronutrient depletion from prolonged exposure	Nonselective transport through membranes eliminates toxins and essential blood constituents. Effluent losses of small water-soluble molecules	Known: Generally, superior hemodynamic stability in critically ill patients; excessive ultrafiltration can provoke hypotension	Hemodynamics: Low-Moderate (caution with dosing and UF) Biocompatibility: High Metabolic: Moderate-High (from prolonged treatment exposure) Others: Hypothermia, electrolyte imbalance	Critically ill patients with hemodynamic instability or severe fluid overload might benefit most; should be aware of electrolyte and metabolic derangements with prolonged use.
PD	Peritoneal membrane injury and inflammation, metabolic and access complications	Exposure to bio-incompatible dialysate induces inflammation. PD-related infection, Mechanical complication from high abdominal pressure	Known: Minimal direct systemic circulatory stress. Risk of peritoneal fluid leak (including hydrothorax) Probable: high intra-abdominal pressure may cause secondary mechanical hemodynamic compromise	Hemodynamics: Low Biocompatibility: Low Metabolic: Moderate-High (hyperglycaemia, protein losses) Others: Infection, high intrabdominal pressure, access-related complications	Lowest risk in hemodynamics but risk of access-related complications

CRRT, continuous renal replacement therapy; HD, hemodialysis; MAP, mean arterial pressure; PD, peritoneal dialysis; RRT, renal replacement therapy; UF, ultrafiltration.

**Table 3:** Selected clinical studies informing RRT modality and prescription strategies in AKI-D.

Study	Design/population	Comparison of RRT modalities	Mortality outcome	Kidney recovery outcome	Key interpretation
**RRT modalities**					
Ye et al. [117]	Systematic review and network meta-analysis of RRT modalities in critically ill patients with AKI	CRRT, intermittent hemodialysis, versus other modalities	No consistent mortality advantage of one modality	Renal recovery outcomes varied across studies	No single extracorporeal modality showed consistently superiority; comparisons are limited by heterogeneity and confounding by indication.
Wald et al. [18]	Secondary analysis of STARRT-AKI	CRRT versus intermittent hemodialysis as initial RRT modality	After balancing baseline characteristics, initial receipt of CRRT was associated with a lower risk of death or RRT dependence at 90-days compared with initial receipt of intermittent hemodialysis. This association was predominantly driven by a lower risk of RRT dependence at 90-days (OR: 0.61; 95% CI, 0.39–0.94).		Modality comparisons remain vulnerable to confounding by indication.
Gabriel et al. [62]	Randomized trial in patients with AKI	High-volume PD versus daily hemodialysis	Similar survival	Shorter treatment duration and faster renal recovery in the PD group	Acute PD may be feasible in selected patients and experienced centers.
Al-Hwiesh et al. [63]	Prospective randomized study in critically ill patients with AKI	Tidal PD versus CVVHDF	Comparable or improved short-term survival in the PD group	Shorter RRT duration and faster renal recovery in the tidal PD group	Findings support feasibility of PD in selected ICU patients, but generalizability depends on expertise and patient selection.
Parapiboon et al. [64]	Multicenter open-label randomized noninferiority trial in severe AKI	Lower-dosage acute PD versus intermittent hemodialysis	No significant difference in 28-day dialysis-free survival	No significant difference in 28-day kidney recovery	Acute PD was not inferior in selected severe AKI patients, supporting PD as a feasible alternative rather than a universally superior modality.
**Timing of initiation of RRT**					
STARRT-AKI [16]	Multicenter randomized trial in critically ill patients with severe AKI	Accelerated versus standard RRT initiation	Accelerated initiation did not reduce 90-day mortality	Higher dialysis dependence at 90 days in the accelerated strategy	Avoiding unnecessary early RRT exposure may be important when urgent indications are absent.
**RRT intensity**					
Wang et al. [72]	Systematic review and individual patient data meta-analysis	Higher- versus standard-intensity RRT	Higher intensity did not improve survival	Higher intensity did not improve dialysis independence	Standard-intensity RRT is generally preferred; higher dose should not be used solely to promote recovery.
Vijayan et al. [73]	Secondary analysis of the ATN study	More frequent versus less frequent intermittent hemodialysis	No clear survival advantage	More frequent dialysis was associated with poorer renal recovery	Excessively frequent intermittent dialysis may increase treatment exposure without improving recovery.
**RRT discontinuation**					
Liu et al. [95]	Multicenter randomized trial in AKI-D after ICU RRT	Conservative indication-triggered dialysis versus conventional thrice-weekly dialysis	Mortality effect uncertain	More dialysis-free days and numerically higher recovery at discharge in conservative arm	Daily reassessment and avoidance of unnecessary ongoing dialysis may reduce treatment exposure.

AKI, acute kidney injury; AKI-D, acute kidney injury treated with renal replacement therapy; ATN study, acute renal failure trial; CRRT, continuous renal replacement therapy; CVVHDF, continuous veno-venous hemodiafiltration; ICU, intensive care unit; PD, peritoneal dialysis; RRT, renal replacement therapy.

### Intermittent HD

With intermittent HD, equivalent net ultrafiltration and clearance targets can be achieved with higher ultrafiltration rates and faster solute removal compared to continuous or prolonged therapies. This characteristic “hemodynamic and osmotic swing” pattern explains why intermittent HD is particularly prone to intradialytic hypotension, in particular during critical illness and in patients with limited cardiovascular reserve [20]. In intermittent HD, dialytrauma frequently presents clinically as intradialytic hypotension, cramps, nausea, headache, chest discomfort, or postdialysis fatigue, reflecting acute circulatory stress. Baseline characteristics such as autonomic dysfunction, cardiac reserve, vascular stiffness, intravascular volume status, and medications (i.e. antihypertensives) significantly impact this risk [20].

Based on studies in chronic dialysis, recurrent intradialytic hypoperfusion is associated with loss of residual kidney function. In a CT perfusion study, renal perfusion decreased by ∼18.4% during a standard HD session and correlated with concurrent myocardial injury [25]. Similar results were reported in other observational analyses, reinforcing ultrafiltration stress as a modifiable contributor to residual renal function loss [51, 52]. These findings support strategies that reduce abrupt hemodynamic stress, such as avoiding excessive ultrafiltration rates, continuously reassessing target fluid removal, and considering alternative modalities in hemodynamically vulnerable patients.

### Continuous renal replacement therapy

CRRT is often selected for hemodynamically fragile patients to enable more gradual fluid and solute control. However, hemodynamic instability can still occur, especially in case of excessive ultrafiltration, severe endothelial dysfunction, and reduced cardiac function [53, 54]. In a multicenter study, commencement of CRRT was associated with an abrupt, ∼50% reduction in urine output [55]. This was only weakly explained by changes in fluid balance, raising the possibility that CRRT exposure combined with changes in fluid and hemodynamic management may contribute to early suppression of diuresis and nonrecovery. Related work suggests that higher net ultrafiltration may interact with urine output changes early after CRRT initiation [56, 57]. Randomized trials investigating RRT timing strategies (e.g. STARRT-AKI) have shown that an accelerated initiation strategy did not improve 90-day mortality but was associated with a higher risk of dialysis dependence at 90 days [16]. These data, together with studies showing that delayed fluid removal is harmful, suggest [58] that overly early or unnecessarily prolonged RRT exposure may increase cumulative treatment exposure without improving patient-centered outcomes after AKI.

Loss of electrolytes and nutrients is common in CRRT [42, 59]. A 2024 meta-analysis showed that hypophosphatemia during CRRT was associated with ∼80 h longer duration of mechanical ventilation and longer ICU stay, but there was no association with mortality [60]. Similar results were observed in secondary analyses of the RENAL trial, pointing out that higher-intensity CRRT produced more days with hypophosphatemia, which was associated with lower chances of successful extubation within 28 days [61]. However, there is no evidence that correcting these losses improves kidney recovery.

### Sustained low-efficiency dialysis and prolonged intermittent RRT

Sustained low-efficiency dialysis (SLED), also referred to as prolonged intermittent RRT (PIRRT), represents an intermediate approach between conventional intermittent HD and CRRT. By extending treatment duration and reducing hourly solute and fluid shifts, SLED/PIRRT may improve hemodynamic tolerance compared with standard intermittent HD while avoiding continuous extracorporeal exposure. This may be particularly useful in patients who are hemodynamically vulnerable but do not require 24-h CRRT. However, evidence that SLED/PIRRT improves renal recovery compared with CRRT or intermittent HD remains limited, and treatment effects are difficult to separate from patient selection, local protocols, staffing models, and anticoagulation practices.

### Peritoneal dialysis

PD avoids extracorporeal blood exposure, blood–membrane interactions, excessive ultrafiltration, and the need for vascular access, and may better preserve hemodynamic stability and renal perfusion. Several randomized studies have evaluated the role of acute PD in patients with AKI. High-volume PD has been compared with daily HD, and tidal PD has been compared with CRRT [62, 63]. These studies reported comparable survival, with signals toward shorter treatment duration, faster renal recovery, and fewer infectious complications in PD-treated patients. More recently, a multicenter, open-label randomized noninferiority trial directly compared lower-dosage acute PD with intermittent HD for severe AKI [64]. There was no significant difference in 28-day dialysis-free survival [42% versus 37%, risk difference 4.6 (95% CI, −11.1 to 20.3)] and 28-day kidney recovery [39% versus 26%, risk difference 12.7 (95% CI, −2.8 to 28.2)]. Taken together, these studies support PD as a feasible option in selected patients. Importantly, acute PD is also associated with adverse events, including elevated intra-abdominal pressure and risk of peritonitis and peritoneal leak, [65] but the risks are lower in short-term acute PD use for AKI-D. Overall, acute PD is used less often than CRRT or intermittent HD, mainly due to the urgent need for a PD catheter and overall limited expertise and familiarity among healthcare providers.

## STRATEGIES TO PROMOTE RENAL RECOVERY DURING RRT

RRT is unlikely to directly accelerate intrinsic renal repair, but it can be delivered in ways that either support or hinder recovery. A recovery-oriented strategy aims to provide adequate control of fluid, electrolyte, and uremic complications while minimizing dialytrauma and unnecessary treatment exposure. This includes careful consideration of potentially modifiable components of the RRT prescription, including RRT timing, modality, and dose, as well as fluid and hemodynamic management [66]. Although no RRT prescription has been shown to directly accelerate intrinsic renal repair, RRT should be delivered in a way that promotes renal recovery. Individualized ultrafiltration targets and close coordination with vasopressor and ventilator management are important [54]. Guideline statements support the use of continuous therapies in hemodynamically unstable patients, reflecting the potential for smoother fluid/solute shifts and fewer abrupt blood pressure drops [67]. The key components of a recovery-oriented RRT prescription are summarized in Box 1.

### Hemodynamic stability during RRT

Hemodynamic instability and acute reductions in systemic blood pressure are considered harmful to renal function and associated with reduced chances of renal recovery. However, the optimal blood pressure target during RRT is not known and likely to be influenced by the patient’s comorbidities and premorbid blood pressure.

Mitigation of intermittent HD-related dialytrauma focuses on reducing the amplitude of circulatory stress while maintaining adequate clearance. Lowering treatment intensity through longer sessions, lower ultrafiltration rates, and more precise dry-weight assessment reduces the risk of hemodynamic instability. Adjunctive strategies include the use of cooler dialysate protocols (36°C) to promote peripheral vasoconstriction and improve vascular tone [25, 68]. Although less notable than hemodynamic instability, blood–membrane bioreactivity may add inflammatory and thrombogenic load and potentially increase the longer-term cardiovascular risk [69].

### Volume management during RRT for AKI

Fluid management during acute RRT is different compared to maintenance chronic dialysis in CKD, where ultrafiltration is often pushed to achieve a “dry weight” during a 3–6-h session. In AKI-D, the priority is metabolic control and early removal of excess fluid with avoidance of iatrogenic hypovolemia and dialysis-related hypotension. Care should be taken to ensure that the ultrafiltration rate does not exceed the plasma refilling rate. In patients on invasive hemodynamic monitoring, trends suggesting intravascular depletion, such as increasing stroke volume variation, should prompt consideration to reduce the ultrafiltration rate. In more stable patients, relative blood volume monitoring may be useful, though more data is needed [70].

### Dialysis modality and dose

Comparative studies of CRRT versus intermittent HD have not demonstrated consistent differences in mortality [71]. Some studies concluded that CRRT may be associated with higher renal recovery compared with intermittent HD, but results are confounded by indication. Substantial heterogeneity exists across studies, but no modality has consistently demonstrated better renal recovery. Selected clinical studies informing RRT modality and prescription strategies in AKI-D are summarized in Table 3.

Current evidence indicates that higher-intensity solute clearance (prescribed dose of 35–48 ml/kg/h) offers no survival or renal recovery advantage over standard-intensity regimens (typically 20–25 ml/kg/h). In fact, higher-intensity RRT may delay renal recovery [72]. Likewise, increasing the frequency of intermittent HD from three to six sessions per week may be linked to poorer renal recovery [73]. Whether reducing RRT intensity below the current standard dose promotes renal recovery is unknown and currently tested formally in RCTs. PD is a feasible option for some patients with AKI-D and might support renal recovery by avoiding the hemodynamic stress of extracorporeal therapy. A multicenter, open-label randomized noninferiority trial directly compared acute PD with intermittent HD for severe AKI [64]. There was no significant difference between the PD and HD groups in 28-day dialysis-free survival [42% versus 37%, risk difference 4.6 (95% CI, −11.1 to 20.3)] and 28-day kidney recovery [39% versus 26%, risk difference 12.7 (95% CI, −2.8 to 28.2)].

### Kidney supportive care

Kidney recovery-supportive care includes reduction of avoidable treatment interruptions and prevention of complications. Appropriate vascular access and effective anticoagulation are necessary to reduce the risk of unplanned interruptions and prolonged periods of fluid overload [74, 75]. Membrane biocompatibility and adsorption properties may also influence inflammatory and complement activation, but whether membrane selection improves renal recovery in AKI-D remains uncertain.

Evidence that active correction of anemia improves kidney recovery is limited. The multicenter pragmatic EAKI trial of recombinant erythropoietin versus usual care in hospitalized patients with AKI and Hb <11 g/dl found no reduction in transfusion requirements and no difference in renal recovery or mortality [76]. Similarly, retrospective data in AKI-D showed no difference in renal recovery, including following administration with erythropoietin [77, 78]. However, trials powered to test renal recovery as a primary endpoint in AKI-D after anemia correction are still lacking.

### Medication and nutrition management

Daily medication review to optimize dosing and limit nephrotoxin exposure is recommended to support renal recovery in AKI guidance, but there are no specific medications to initiate renal recovery [79, 80]. In patients with CKD, use of renin-angiotensin-aldosterone system inhibitors (RAASi) is linked with improved renal outcomes, mainly as a result of a reduction of glomerular pressure, control of blood pressure, and attenuation of inflammation and oxidative stress. There is evidence from observational studies that reinitiating or newly starting RAASi therapy after AKI, is associated with lower risks of all-cause mortality, recurrent AKI and progression to incident CKD [81]. Similarly, sodium-glucose cotransporter-2 (SGLT2) inhibitors reduce intraglomerular pressure and have anti-fibrotic effects, thereby offering renoprotection. A large retrospective cohort study from Taiwan reported lower risk of all-cause mortality and major adverse kidney events with the use of SGLT2 inhibitors in people with diabetes and acute kidney disease and clinical indications for restarting treatment [82]. However, the optimal timing to restart or initiate these medications during or after AKI-D is unknown [83, 84]. Results of ongoing trials are awaited.

There is little evidence that any specific feeding strategy accelerates renal recovery [85]. A prespecified analysis of the “Early Parenteral Nutrition Completing Enteral Nutrition in Adult Critically Ill Patients” (EPaNIC) study showed that the timing of parenteral nutrition did not affect the incidence of AKI, but early initiation slowed renal recovery in patients with stage 2 AKI [86]. Early parenteral nutrition did not affect the time course of creatinine and creatinine clearance but did increase plasma urea, urea/creatinine ratio, and nitrogen excretion beginning on the first day of amino acid infusion. Current guidelines focus on providing adequate (but not excessive) calories and protein, adjusted for illness severity and RRT-related losses [87].

### Dialysis discontinuation

Unnecessary prolongation of dialysis exposure may itself contribute to nonrecovery through cumulative dialytrauma. As patients stabilize, transition from continuous to intermittent modalities may be considered, shortening the duration of sessions and/or reducing treatment frequency. It is often unclear when kidney function has recovered enough to remain dialysis-free after stopping RRT. Urine output during RRT appears to be the best marker, but the exact threshold predicting successful versus unsuccessful RRT discontinuation varies between studies [88]. While prolonged RRT exposure increases treatment burden and the risk of dialytrauma, discontinuing RRT prematurely can leave patients fluid overloaded and uremic for longer.

Since the transition from dialysis dependence to independence is often gradual, structured daily reassessment for discontinuation is essential, including comparison with predialysis serum creatinine results, timed urine collection to measure creatinine clearance, and evaluation of blood pressure and volume status. Declining predialysis serum creatinine values without changes in RRT prescription may signal renal recovery. However, if urine output is not increasing, creatinine trends should be interpreted cautiously, as decreases can reflect illness-related reduction in creatinine generation. Dialysis transiently suppresses native urine output and renal solute handling. To capture residual kidney function as accurately as possible, a 6–24-h urine collection should be obtained during the interval before the next HD session, together with a predialysis blood sample [89]. The kidney function threshold required to safely stop RRT is not firmly defined, but a creatinine clearance <11 ml/min is likely insufficient [90, 91].

The literature on weaning from acute RRT consistently identifies urine output as the most commonly used and most studied indicator in practice, while also acknowledging that no single threshold is universally reliable and heterogeneity across studies exists [92, 93]. Patients who produce >400 ml/day of urine without diuretics, or >2300 ml/day with diuretics, have an >80% likelihood of successfully stopping CRRT [94].

To address when dialysis can be stopped safely, the multicenter RCT LIBERATE-D assigned adults with AKI who had had at least one session of CRRT or intermittent HD during their stay in the ICU to either a conservative, indication-triggered reinitiation strategy or conventional thrice-weekly intermittent HD until prespecified urine output or creatinine clearance criteria were met [95]. All participants were hemodynamically stable without needing vasopressor support. Patients randomized to the conservative strategy had a better chance of recovery of renal function by the time of hospital discharge (64% versus 50%), needed fewer dialysis sessions per week, and had more consecutive dialysis-free days by day 28. In addition, dialysis-associated hypotension events were less frequent. However, the differences were statistically no longer significant when adjusting for other factors, underscoring uncertainty about the true effect size and the need for confirmatory studies.

## INDICATORS OF RENAL RECOVERY

Serum creatinine and urine output remain the most widely used measure of kidney function during and after AKI-D but have important limitations [11, 96, 97]. Even if serum creatinine returns to baseline following AKI-D, this may not assure renal recovery or good kidney health [98]. Based on observational studies, a urine output >400 ml/24 h without exposure to diuretics or >2300 ml/24 h following exposure to diuretics and timed creatinine clearance can be used to predict successful discontinuation of RRT [88]. The ability to concentrate urea reflects tubular cell health and medullary integrity, and urea clearance has been reported as a marker of tubular function. Whether urea clearance can guide the dialysis weaning phase needs to be investigated in future research.

Alternative kidney biomarkers of persistent AKI/nonrecovery have also been investigated but have not been integrated into routine clinical practice. In 331 critically ill patients with AKI stage 2 or 3, the urinary biomarker CCL14 identified those with a high risk of delayed renal recovery [99]. A meta-analysis of six studies including 952 patients with AKI concluded that urinary CCL14 was an effective marker for predicting persistent AKI with a pooled diagnostic accuracy of 0.84 [100]. Similarly, a study evaluating the role of proenkephalin A in sepsis/septic shock showed that the proenkephalin levels within 24 h of ICU admission were significantly higher in those patients who subsequently developed persistent or worsening AKI [101]. Finally, in 733 patients undergoing cardiac surgery, a preoperative ratio of urinary Dickkopf-3 (DKK3) to creatinine concentrations >471 pg/mg was associated with a significantly higher risk of persistent kidney dysfunction and dialysis dependency (OR: 13.57; 95% CI, 1.50–122.77, *P* = .020) after 90 days compared with a ratio of 471 pg/mg or less [102]. However, although potentially predictive of worsening AKI, none of these biomarkers have been found useful to guide discontinuation of RRT [88].

## CONSIDERATIONS IN SPECIFIC PATIENT POPULATIONS

### Patients with advanced CKD

Patients with advanced CKD, defined as eGFR <30 ml/min/1.73 m^2^, have a higher risk of developing AKI-D and progressing to end-stage renal disease (ESRD) [103–105]. While baseline eGFR is a strong predictor of eventual RRT independence, a small proportion of patients with advanced CKD may still recover sufficient renal function to wean off dialysis. In most patients, resolution of AKI occurs within the first three months, though up to 25% continue to improve beyond this traditional timeframe [106]. However, the majority of patients with AKI-D on the background of advanced CKD do not recover renal function back to baseline. They should still be monitored closely for potential renal recovery, as unwarranted RRT prolongation results in increased dialysis dependence and mortality [72].

Once the cause of AKI has resolved and the patient is clinically improving with increasing urine output, RRT discontinuation should be attempted with consideration of reintroduction of medical therapy, such as potassium binders and sodium bicarbonate. In case of persistent fluid and/or metabolic derangements despite maximal medical therapy, RRT reinitiation is warranted [72].

Shared decision-making is necessary for all patients, in particular those with advanced CKD, and includes discussions about the probability and expected timeframe of kidney recovery, therapeutic options if recovery does not occur, and overall goals of care [107].

### Patients with congestive heart failure

Key aspects of RRT in patients with congestive heart failure include proper volume assessment and avoidance of hemodynamic instability. Patients often suffer from both, left- and right-sided heart failure, impairing renal recovery [108]. Volume assessment with clinical examination and objective tools, such as point-of-care ultrasound, is crucial to avoid inadequate perfusion and venous congestion [109].

While the choice of acute intermittent HD versus CRRT does not appear to influence survival or subsequent renal recovery, CRRT offers better cardiovascular stability and more controlled solute and fluid removal [67]. CRRT also prevents fluctuation in fluid status. In hemodynamically stable patients, intermittent HD can be used. However, care should be taken to mitigate intra-dialytic hypotension and prevent further renal ischemia. PD is a reasonable alternative for hemodynamically stable patients.

In diuretic-resistant heart failure without significant metabolic derangement, isolated ultrafiltration can be used for decongestion. This may provoke less neurohormonal activation than diuretics. However, the evidence is conflicting [53, 110–113].

### Very elderly patients

Patients who are very elderly, typically defined as >80 years of age, are at higher risk of hemodynamic instability during RRT, mainly due to concomitant cardiac disease and related medications. On the other hand, they often have decreased thirst sensation, a lower metabolic demand, and a lower creatinine generation rate, which means that lower RRT intensity and lower ultrafiltration rates may suffice. The RRT prescription should account for baseline functional status, likelihood of kidney recovery, and overall goals of care, with particular attention to avoiding complications from unnecessary or overly intensive RRT.

### Kidney transplant recipients

There is very little clinical guidance in the literature for this group of patients [114, 115]. Recommendations related to RRT are largely extrapolated from general AKI-D principles and should be individualized according to graft function, immunologic risk, infection, hemodynamic status, and exposure to nephrotoxic immunosuppressive drugs [114]. A pooled analysis of four studies in kidney transplant recipients with delayed graft function found no effect of membrane biocompatibility on renal recovery (RR: 1.05; 95% CI, 0.87–1.26) [116]. Modern dialysis practice predominantly uses synthetic membranes with improved biocompatibility, although available membrane materials still differ in the degree of complement and inflammatory activation they induce [33].

## CONCLUSIONS

The “best” dialysis regimen for AKI-D depends on the clinical context, but the core principles to promote renal recovery are to use the least intensive dialysis necessary to control complications, to avoid nephrotoxic exposures and to monitor closely for signs of resolution of AKI [84] (Table 4 and Fig. 2). Further research is urgently needed to inform individualized RRT prescriptions that reduce dialytrauma and support long-term kidney health after AKI-D [96].

**Figure 2:**
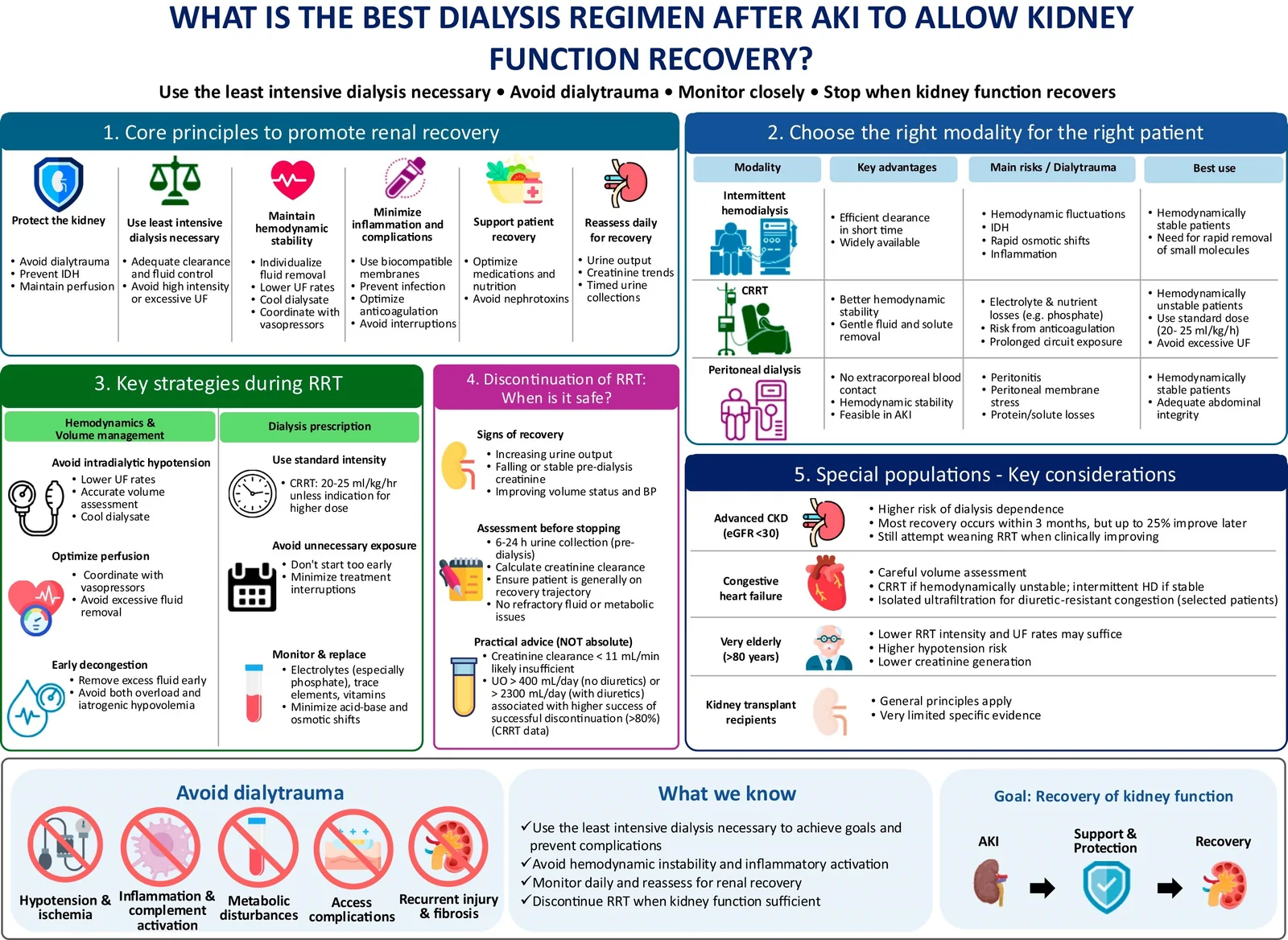
What is the best dialysis regimen after AKI to allow kidney function recovery? The figure lists the core principles to promote renal recovery and outlines strategies during renal replacement therapy. Key considerations in specific high-risk groups are highlighted.

**Table 4:** Daily assessment for RRT de-escalation or discontinuation in AKI-D.

Domain	Assessment	Clinical implication
RRT indication	Persistent urgent indication: hyperkalemia, acidosis, uremia, fluid overload	Continue RRT if not controlled
Urine output	Increasing urine output or lower diuretic need	Suggests native kidney recovery
Solute control	Potassium, urea/BUN, creatinine trajectory	Assess ability to remain off RRT
Acid-base status	pH, bicarbonate, lactate trend	Ensure metabolic stability
Volume status	Actual and expected fluid input, urine output, oxygenation, edema/congestion	Assess whether fluid balance can be maintained without RRT
Residual clearance	Timed CrCl or average CrCl/UreaCl	Estimate native kidney clearance
Hemodynamic stability	BP trend, vasopressor need, perfusion markers	Determine safety of de-escalation
Overall trajectory	nutrition, anticipated procedures, goals of care	Contextualize timing of liberation

AKI-D, acute kidney injury treated with renal replacement therapy; BP, blood pressure; BUN, blood urea nitrogen; CrCl, creatinine clearance; UreaCl, urea clearance; RRT, renal replacement therapy.

## Data Availability

No new data were generated or analyzed in support of this research.
